# Synthesis and Supramolecular Assembly of a Terrylene Diimide Derivative Decorated With Long Branched Alkyl Chains

**DOI:** 10.3389/fchem.2019.00473

**Published:** 2019-07-03

**Authors:** Zongxia Guo, Xiao Zhang, Lu Zhang, Yujiao Wang, Weisheng Feng, Kai Sun, Yuanping Yi, Zhibo Li

**Affiliations:** ^1^Key Laboratory of Optic-electric Sensing and Analytical Chemistry for Life Science, MOE, Key Laboratory of Biochemical Analysis, Shandong Province, College of Chemistry and Molecular Engineering, Qingdao University of Science and Technology, Qingdao, China; ^2^Key Laboratory of Biobased Polymer Materials, Shandong Provincial Education Department, School of Polymer Science and Engineering, Qingdao University of Science and Technology, Qingdao, China; ^3^Institute of Chemistry, Chinese Academy of Sciences, Beijing, China

**Keywords:** terrylene diimides, supramolecular assembly, scanning tunneling microscope, aromatic systems, liquid-solid interface

## Abstract

Terrylene diimide derivatives are pigments for dyes and optoelectric devices. A terrylene diimide derivative N,N'-di(1-undecyldodecyl)terrylene-3,4:11,12-tetracarboxdiimide (DUO-TDI) decorated with long branched alkyl chains on both imide nitrogen atoms was designed and synthesized. The supramolecular assembly behaviors of DUO-TDI in solution and at the liquid-solid interface were both investigated. The assembled nanostructures and photophysical properties of TDI in solution were explored by varying solvent polarity with spectral methods (UV-Vis, FL and FT-IR) and morphological characterization (AFM). Depending on the solution polarities, fibers, disk structures and wires could be observed and they showed diverse photophysical properties. In addition, the interfacial assembly of DUO-TDI was further investigated at the liquid-Highly Oriented Pyrolytic Graphite (HOPG) interface probed by scanning tunneling microscope (STM). Long range ordered monolayers composed of lamellar structures were obtained. The assembly mechanisms were studied for DUO-TDI both in solution and at the interface. Our investigation provides alternative strategy for designing and manipulation of supramolecular nanostructures and corresponding properties of TDI based materials.

## Introduction

Rylene diimide dyes derivatives are famous for their outstanding photophysical and photochemical stability and their high fluorescence quantum yield (Zhao et al., 2016; Feng et al., 2017; Frankaer et al., 2019). They have not only shown importance as vat dyes in industrial olorants, but also have been proven to be excellent organic semiconductor candidate for opto-electronic applications (Geerts et al., 1998; Wolf-Klein et al., 2002; Jung et al., 2006). Terrylene diimides (TDIs) are a class of rylene diimide dye consisting of terrylene core, a large aromatic core along the long molecular axis. It shows brilliant blue color and emits fluorescence at long wavelengths with long fluorescence lifetime. Moreover, TDIs also show good thermal, chemical, and photochemical stabilities. TDIs are potential candidates as excellent probes for bio-labeling, energy convertors for light concentrators, and functional materials in electronic devices (Peneva et al., 2008; Bai et al., 2011; Berberich and Würthner, 2012; Chen et al., 2014; Stappert et al., 2016). Then, the design and synthesis of TDI based molecules have been attracting increasing attention recently, although less research had been done compared to perylene diimides (PDIs) (Chen et al., 2015; Würthner et al., 2016; Guo et al., 2019), another kind of rylene diimides. TDI derivatives were mostly designed via the decoration of the parent TDI molecule with various functional groups on the imide positions or on the periphery of the terrylene core (Heek et al., 2013). Actually, the competition and cooperation of the π-π stacking from the terrylene cores and other weak interactions of added functional groups play a great role in the modulation of molecular packing and their nanostructures and properties. One of the strategies is by using flexible chains, especially the alkyl chains (Davies et al., 2011). The affiliation of alkyl chains could vary the solubility, processing ability, molecular arrangement way, and the corresponding properties of TDIs. The topological structures of the alkyl chains could affect the assembly of rylene imides as well (Balakrishnan et al., 2006). It was proved that branched alkyl chains were capable of promoting distinguished assembly than induced by normal alkyl chains (Liao et al., 2013). For TDIs, branched alkyl chains were indeed fixed on the aromatic core to study the assembly therefore (Nolde et al., 2006). It should be known that the length of the alkyl chains could greatly affect the molecular assembly even with only one methylene difference (Chesneau et al., 2010; Xu et al., 2013; Li et al., 2017). Here, we report on the synthesis and self-assembly of one terrylene imide derivative modified with alkyl chains. For this subjective, it has two branched long alkyl chains on both imide positions. The presence of long alkyl chains significantly enhances the solubility and inhibits the intermolecular interaction and aggregation. The modulation of the solution assembly of TDI was realized by changing the solvent polarity and monitored by spectral and morphological methods. It was found that different kinds of assembled nanostructures with various properties could be formed. In addition, the surface/interfacial assembly behaviors could provide insights into the design, select and optimizing of semiconductors for using in opto-electronic devices. Then the assembly of TDI at the liquid-HOPG interface was also explored by STM (Lee et al., 2014a,b).

## Results and Discussions

### Synthesis and Properties of DUO-TDI

**Scheme 1:**
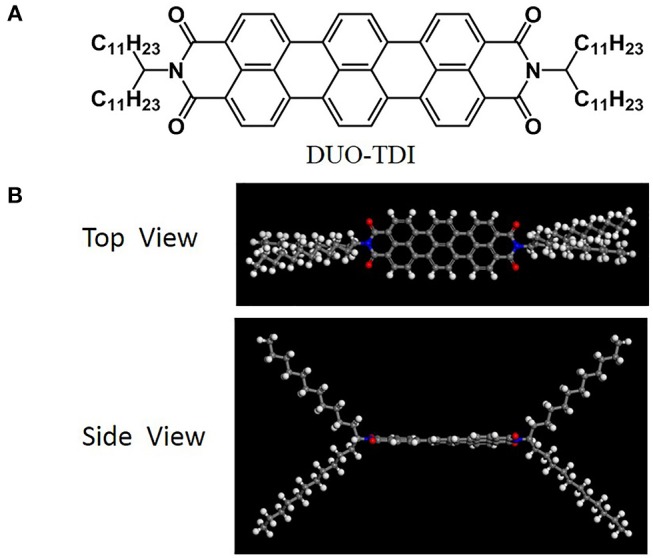
**(A)** Molecular structure of DUO-TDI. **(B)** The top and side view of DUO-TDI molecule. The optimization was performed using the Forcite module of Materials Studio 7.0. The DREIDING force field was implemented for the geometry optimizations (Mayo et al., 1990).

DUO-TDI was synthesized based on reported methods (Mayo et al., 1990; Nolde et al., 2006) (Scheme 1). Blue powder was obtained for DUO-TDI. The molecular structure was confirmed by ^1^HNMR, ^13^CNMR and MALDI TOF MS. As indicated in the Scheme 1, a large aromatic core exists between two branched alkyl chains. From the optimized molecular structure of TDI, the aromatic core is almost planar from the side view of the molecular structure. The distance between the two N atoms in the molecular skeleton is about 1.58 nm and such large π-conjugated core could provide strong π-π interactions with neighboring conjugated systems. The four undecyl chains with length about 1.39 nm in the periphery of the core structure not only change the solubility in usual organic solvents, but also offer van der Waals interactions among adjacent chains. It could be expected that the synergistic effect between π-π interactions from the core and the van der Waals interactions from such long alkyl chains could be effectively modulated by varying the conditions, thus leading to diverse assembly process, assembled nanostructures, and properties (Chen et al., 2015; Zhang et al., 2016).

**Figure 1:**
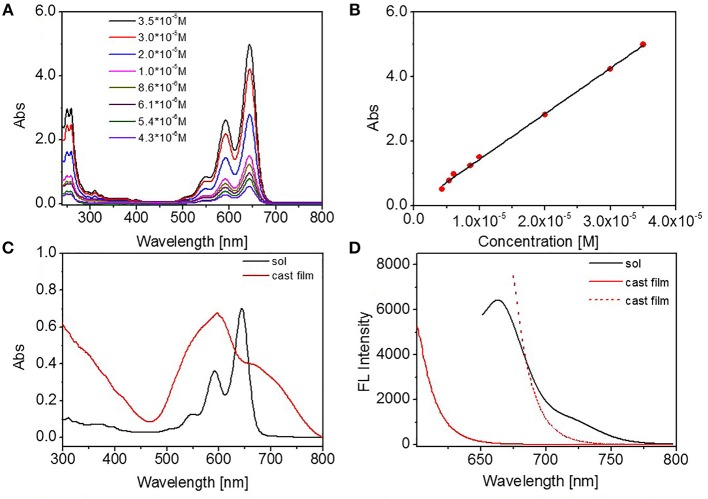
**(A)** Concentration-dependent UV absorbance spectra of DUO-TDI in THF solutions, **(B)** The absorbance intensity at 644 nm as a function of concentration. The fitting R squared factor was 0.9966, **(C)** UV absorption spectra of DUO-TDI in THF solution (1.5 × 10^−5^ M) and cast film, **(D)** FL spectra of DUO-TDI in THF solution (1.5 × 10^−5^ M) and cast film. The excitation wavelength (λex) was 644 nm for solution. And Xex = 598 (red solid line) or 672 nm (red dashed line) for cast film.

Tetrahydrofuran (THF) is a good solvent for DUO-TDI. The absorption and emission properties of DUO-TDI in molecular state were investigated by UV-Vis and FL emission spectra. Firstly, the concentration-dependent UV-Vis absorption spectra of DUO-TDI in THF solutions were studied (Figure 1A). It can be seen that all the UV-Vis absorption spectra exhibited well-resolved vibronic structures when the concentration was varied from 4.3 × 10^−6^ to 3.5 × 10^−5^ M. With the concentration increasing, the absorbance intensity increased accordingly but without band shift. The relationship of absorbance intensity at 644 nm as a function of the concentration was shown in Figure 1B. From the fitted linear line, it was clear that DUO-TDI did not assemble into aggregates. In another words, DUO-TDI exists in molecularly state in THF within the above concentration range. In solution, the absorbance band from 450 to 700 nm was ascribed to the π-π^*^ electronic transition of the chromophores in the monomeric state along with vibrational transitions (Figure 1C). Four characteristic vibration absorption bands centered at 644, 591, 546, and 505 nm were observed, and attributed to the 0-0, 0-1, 0-2, and 0-3 vibrational transitions, respectively (Nagao et al., 2002).

The cast film from THF solution of DUO-TDI was also studied to compare with the molecularly DUO-TDI. Two structureless bands at 670 and 597 nm were obtained for the cast film. The band at 597 nm should shift from that at 644 nm from diluted THF solution. Such blue shift indicated the formation of H-aggregates in the film (Davies et al., 2011). At the same time, the appeared shoulder band at 670 nm suggested the existence of J-aggregates in the cast film (Jung et al., 2006). From FL spectra, one emission peak at 672 nm and a shoulder band at 725 nm showed up for DUO-TDI in diluted solution. In contrast, the emission of DUO-TDI was completely quenched in the cast film, suggesting the main formation of H-type molecular packing in accordance with the results from UV-vis data (Jung et al., 2006).

### Solvent Induced Assembly of DUO-TDI

**Figure 2:**
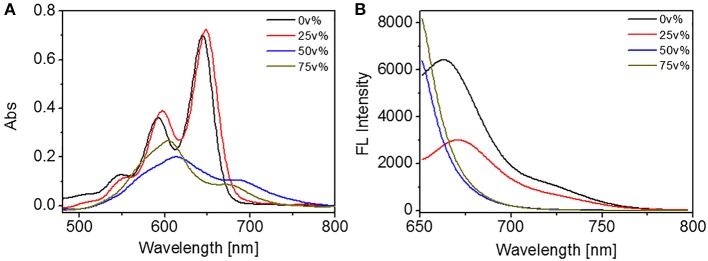
**(A)** UV absorption and **(B)** FL emission spectra of DUO-TDI in solutions of water/THF. The concentration of all mixed solutions was 5.4 × 10^−6^ M. The excitation wavelength was 644 nm for all samples.

Apart from the above discussions on the molecular behaviors in diluted solutions and in films, the self-assembly of TDI was further investigated in mixed solvent with varied polarity. In the present contribution, the mixed solvents were prepared by adding water into THF solutions. The volume percentage of water (Vw, v %) in the mixed solvent was altered to adjust the solvent polarity. Vw was changed from 0 to 75v% to study the solvent-dependent self-assembly of DUO-TDI, which was monitored by UV-vis and FL spectra firstly (Figure 2). With Vw = 25 v%, the absorption spectral lineshape was almost the same to that from monomeric DUO-TDI in THF (0 v%), while the absorbance intensity was slightly enhanced and the bands shifted to red. It can be seen that the three monomeric absorption bands at 546, 591, and 644 nm, which belong to the 0-2, 0-1, and 0-0 electronic transitions from the terrylene diimide cores red-shifted to 549, 597, and 648 nm. FL spectra were recorded to shed light on the self-assembly of DUO-TDI. It was shown that the emission was quenched greatly (about 50%) and the main emission band at around 662 nm red-shifted to 670 nm. It could be concluded that J-aggregates were formed with Vw = 25 v% (Jung et al., 2006). When Vw was raised to 50 v%, drastic changes of absorption band were observed. The absorption bands were broadened and turned into unresolved structures. Two main bands appeared at 600 and 690 nm, accompanied by incremental absorption at a wavelength longer than 700 nm. Clearly, the absorption at 612 nm was blue-shifted from the band at 644 nm, and the absorption at 690 nm was a newly appeared band. In addition, the emission was quenched as well. It was demonstrated that H-aggregates were mainly formed with J-aggregates in a minority in solution with Vw = 50 v%. With Vw = 75 v%, it showed similar spectral lineshape to that of 50 v%, however the two main peaks were centered at 600 and 680 nm (Figure 2A). It was obvious that the blue shift was enlarged compared to that from solution with Vw = 50 v%, indicating the increased π-π stacking. Apart from that, the relative intensity at about 600 and 690 nm was increased from 1.89 to 3.14, suggesting the increased relative amount of H- to J-aggregates. Besides, the fluorescence emission was completely quenched in accordance with the results from UV-vis spectra. Based on the above results, DUO-TDI could assemble into J- or H-type of aggregates relying on the solvent condition. Slightly increasing polarity of solvent, J-aggregates would be formed, and the elevation of polarity could facilatate the formation of H-aggregates. The molecular arrangement of DUO-TDI within the aggregates could be altered by changing solvent polarity.

**Figure 3:**
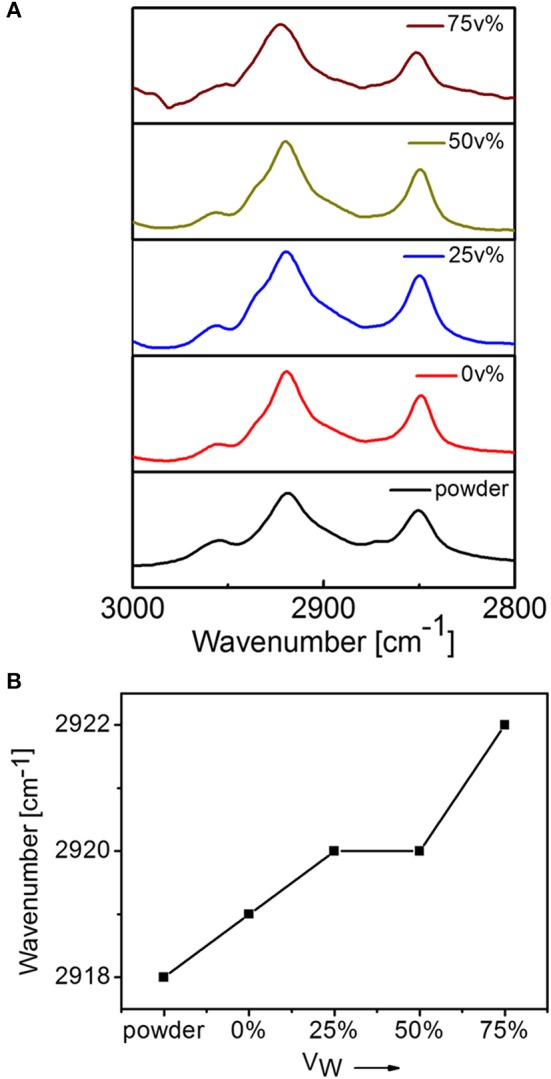
**(A)** FT-IT spectra of DUO-TDI in solutions with variable V_w_ (0, 25, 50, and 75 v%). **(B)** Variation of peak positions of asymmetric stretching vibration of CH_2_.

FT-IR spectral method was used to detect alkyl chain packing in the molecular assemblies (Figure 3). It was reported that the alkyl chains with all trans-cis zigzag conformation could show an asymmetric stretching vibration of methylene group (CH_2_) at 2,916–2,918 cm^−1^ (Wang et al., 1996; Zhang et al., 1997). For DUO-TDI powder, the asymmetric stretching vibration was at 2,918 cm^−1^, which indicated all trans-cis zigzag conformation of alkyl chains. It was found that this peak shifted on varying V_W_. The absorption of CH_2_ from the branched undecyl groups were at 2,919 cm^−1^ from THF, indicating the relative disordered packing of alkyl chains. With the increase of polarity by the addition of water, the asymmetric vibrations of CH_2_ shifted to longer wavenumbers, from 2,918 to 2,922 cm^−1^, implying that gauche conformation or disordered packing of alkyl chains increased gradually. From the FT-IR and UV-Vis spectral data, both the π-π stacking and the alkyl chain packing were both varied on changing the solvent conditions.

**Figure 4:**
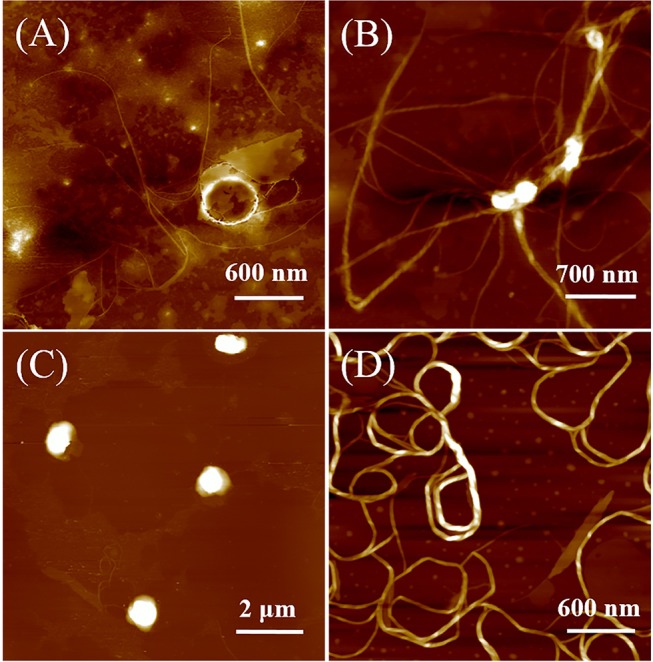
AFM images of DUO-TDI films casted from solutions with varied Vw. **(A)** Vw = 0 v%, **(B)** Vw = 25 v%, **(C)** Vw = 50 v %, and **(D)** Vw = 75 v%. The concentration of all mixed solutions was 5.4 × 10^−6^ M.

Atomic force microscopy (AFM) measurements were carried out to investigate the polarity effect on the self-assembled nanostructures of DUO-TDI. Figure 4 shows the AFM images of DUO-TDI nanostructures formed in different mixed solutions. It was evident that in the cast film from pure THF solution, no uniform structures could be observed. Amounts of amorphous structures existed on the surface with few thin fibers. The width of the fibers was around 18 nm. The observed fiber structures might be obtained due to the evaporation of THF on mica surface, since there was no obvious aggregation behaviors were found based on the solution spectral data. For V_W_ = 25 v%, helical fibers with left-handedness were mainly obtained. It means that chiral nanostructures were assembled, although DUO-TDI is an achiral building block (Shen et al., 2014). It was suggested that the J-aggregation manner facilitates DUO-TDI to hierarchically assemble into structures with handedness. The height of the helical fibers was around 13 nm. The width of the fibers was about 70 nm. It can be easily seen that thick fibers were entangled by thin fibers. With V_W_ = 50 v%, helical fibers almost disappeared, and there were sphere structures with diameters ~1 μm and the height about 100 nm. Considering the high ratio (~ 10) of width to height, it could be deduced that actually disk structures were formed. So, the emergence of both H- and J-aggregates could prevent the hierarchical growth of one dimensional fibers and the formation of chiral sense for the nanostructures. On increasing the V_W_ to 75 v%, lots of wires with an average width of 50 nm showed up without obvious helical sense. Since H-aggregation manner was the major way of molecular packing with V_W_ = 75 v%, then it seems that the H-aggregates could promote the growth of one dimensional structures, but inhibit both chiral packing of molecules and hierarchical growth with chirality. It was clear that the solvent polarity indeed affected the assembled structures of DUO-TDI. The formation of helical fibers, disk structures and wires could be manipulated by controlling the solvent polarity and this further confirmed the different molecular packing modes and hierarchical ways within the assembled nanostructures. The AFM data was in accordance with that from the spectral results.

### Assembly of DUO-TDI at the Liquid-HOPG Interface

We also reported the self-assembly of this n-type semiconductor at the liquid-HOPG interface. For DUO-TDI, the large π-conjugated core provides the π-π staking interactions with the substrate; and the four long alkyl chains offer potential good affinity with the HOPG surface and Van der Walls interactions among neighboring alkyl chains (Chen et al., 2014; Liu et al., 2015, 2016). Different solvents were tried, since the solvent may affect the self-assembly behaviors of molecules at the interface between the liquid and HOPG (Shen et al., 2010; Li et al., 2016). Here, 1-Octanoic acid and 1-Phenyloctane were used for detect the solvent effect. 1-Octanoic acid is a polar and protic solvent, and 1-Phenyloctane is an apolar and aprotic solvent.

**Figure 5:**
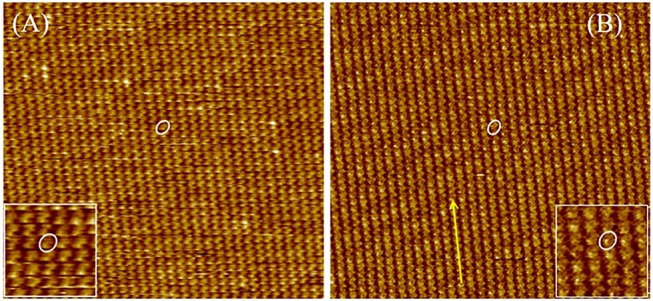
Large-scale STM images (60 × 60 nm^2^) of DUO-TDI at the **(A)** 1-Octanoic acid-HOPG interface and **(B)** 1-Phenyloctane-HOPG interface. Imaging conditions: **(A)***I*_*set*_ = 70 pA, *V*_*bias*_ = −750 mV; **(B)***I*_*set*_ = 75 pA, *V*_*bias*_ = −750 mV. One DUO-TDI is enclosed by a white oval in each image, and the inset indicates the magnified image for clarity. The yellow arrow points to the structure direction of the strip.

In the first stage, the 2D crystallization behaviors of DUO-TDI at 1-Octanoic acid-HOPG interface were investigated. It was found that DUO-TDI could form ordered stable monolayers composed of lamellar structures (Figure 5). Figure 5A showed a large range of monolayers of DUO-TDI molecules. The relative bright dots were attributed to the π-conjugated core of DUO-TDI (Figure S1). Obviously, only two out of four alkyl chains of DUO-TDI adsorbed at the interface. One DUO-TDI core was enclosed by a white oval and enlarged in the inset of Figure 5A for clarity. The one well-ordered lamellar structure was indicated by a yellow arrow. Similar ordered stable monolayers were obtained for DUO-TDI at the 1-Phenyloctane-HOPG interface (Figure 5B).

**Figure 6:**
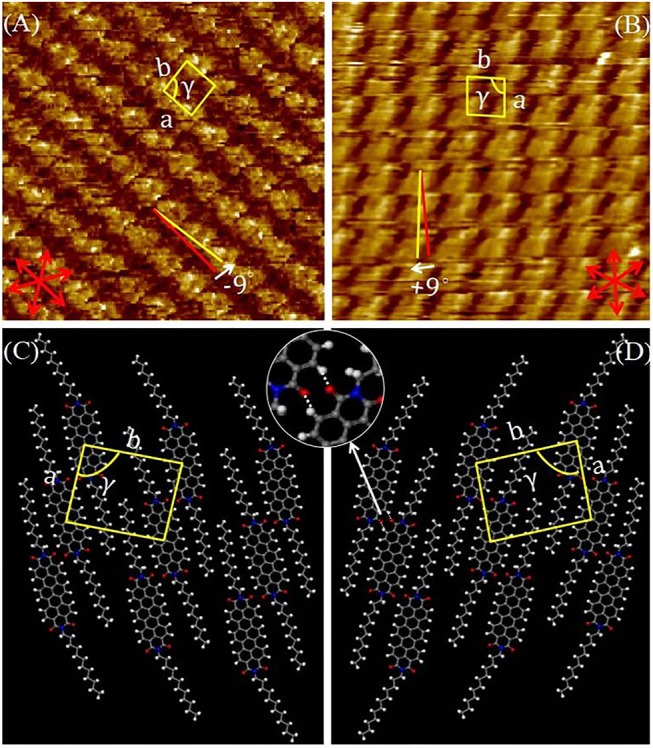
**(A,B)** High-resolution STM images (15 × 15 mm^2^, 4.3 × 10^−6^ M) of DUO-TDI at the 1-Octanoic acid-HOPG interface. Imaging conditions **(A)** *I*_*set*_ = 70 pA, *V*_*bias*_ = −750 mV; **(B)** *I*_*set*_ = 70 pA, *V*_*bias*_ = −750 mV. The graphite symmetry axes are shown in red. The yellow lines indicate the directions of the unit-cell vectors. **(C,D)** Tentative molecular packing models corresponding to the STM images. Hydrogen bonds are indicated by white dotted lines.

To inspect the 2D molecular packing of this semiconductor in more detail, we recorded the high-resolution STM images. Figure 6 showed a high-resolution image of DUO-TDI at the 1-Octanoic acid-HOPG interface. The unit cell parameters of the mirror patterns were the same within experimental error: *a* = 1.53 ± 0.01 nm, *b* = 1.96 ± 0.02 nm, and γ = 86 ± 2° for the packing in Figure 6A; *a* = 1.55 ± 0.02 nm, *b* = 1.97 ± 0.05 nm, and γ = 86 ± 1° for the packing in Figure 6B. In addition, the orientation angles of vector a with respect to the main symmetry axes of the underneath HOPG for the enantiomeric patterns were −9° and + 9° (Figures 6A,B), respectively. Thus, 2D chirality was not only expressed within the monolayer plane, but also at the level of the monolayer orientation with respect to the HOPG substrate (Elemans et al., 2009; Guo et al., 2017). The tentative models for DUO-TDI are shown in Figures 6C,D, where the mirror-related patterns were clearly demonstrated. In rows, two DUO-TDI molecules were aligned in a shoulder-to-shoulder manner to form a dimer, indicated in the zoomed-in images of Figure 6D. Such dimers were connected with each other through two pairs of H-bonds (C-H···O) within the same row. In STM images, the bright rows in the STM image corresponds to the molecular benzene ring skeleton in the model, and the dark rows corresponds to the alkyl chain in the model. The high resolution STM images at the 1-Phenyloctane-HOPG interface were also recorded and the monolayer composed of same nanopatterns with same unit cell parameters was obtained (Figure S2). However, it should be noted that the orientation angle of vector a with respect to the main symmetry axes of the underneath HOPG was 0° for both enantiomeric patterns. It can be seen that DUO-TDI molecules could form same long-term ordered nanostructures at both liquid-HOPG interface, but that the monolayer chirality was changed. Form above discussions, the formation of stable monolayers were attributed by the synergistic effect of H-bonds, π-π stacking, and Van der Walls interactions. And the solvent played an important role in the expression of supramolecular chirality, especially at the level of monolayers.

Actually, the bulk assemblies for DUO-TDI were examined by using the TGA and DSC **(Figure S3)**. TGA was used to characterize the thermal stability, and it was showed that DUO-TDI could be stable at the temperature lower than 360°C. DSC was performed to detect the phase transition of the DUO-TDI molecules. It was found that DUO-TDI showed two peaks at 145 and 137°C in the first cooling curve. It was obvious a thermotropic liquid crystal behavior was observed (reference). The systematic investigation of thermotropic liquid crystal behavior of DUO-TDI is undergoing.

## Conclusions

The synthesis and the investigation of supramolecular assembly of a terylene diimide derivative DUO-TDI have been reported. DUO-TDI has a large π-conjugated core decorated with long branched 1-undecyldodecyl at both N positions. It was found that the supramolecular assembly could be manipulated by changing polarities of solutions consisting good solvent THF and poor solvent water. When varying the volume percentage of water (Vw) from 0 to 75v%, monomeric DUO-TDI, J-aggregates, H-aggregates with minor J-aggregates were obtained. Moreover, J-aggregates benefited for the formation of helical fibers, H-aggregates facilitated the fabrication of achiral nanostructures, such as nanodisks and wires. UV-vis, FL and FT-IR spectra confirmed that π-π stacking and alkyl chain packing were both altered within different nanostructures resulted from the difference in solvent polarity. The assembly of DUO-TDI at the liquid-HOPG interface was also studied. Stable monolayers composed of lamellar structures were observed. Chirality at the pattern level and monolayer level showed up for DUO-TDI at the 1-Octanoic acid-HOPG interface. While the monolayer level chirality disappeared at the 1-Phenyloctane-HOPG interface. The synergetic effect of π-π stacking from the large aromatic core and Van der Walls interactions from alkyl chains was proposed to contribute to the assembly of DUO-TDI in solutions and at the interface. The present investigation provides insight into the design of TDI based semiconductors for both academic research and potential opto-electronic devices or materials.

## Data Availability

The raw data supporting the conclusions of this manuscript will be made available by the authors, without undue reservation, to any qualified researcher.

## Author Contributions

ZG and ZL contributed to the idea of the work and the preparation of the manuscript. XZ, LZ, and YW have done all experiments on the assembly and corresponding analysis. YW, WF, and YY did the simulation work. KS synthesized the molecule DUO-TDI.

### Conflict of Interest Statement

The authors declare that the research was conducted in the absence of any commercial or financial relationships that could be construed as a potential conflict of interest.
