# Supplementary material for: Synthesis and Supramolecular Assembly of a Terrylene Diimide Derivative Decorated With Long Branched Alkyl Chains
Source: Front Chem. 2019 Jul 3;7:473. doi: 10.3389/fchem.2019.00473 (PMC6618297; doi:10.3389/fchem.2019.00473)
Supplement: Supplementary file 1 [file Data_Sheet_1.pdf]

Supplementary Material

Synthesis and Supramolecular Assembly of a Terrylene Diimide Derivative Decorated With Long Branched Alkyl Chains

Zongxia Guo[1*], Xiao Zhang[2], Lu Zhang[2], Yujiao Wang[2], Weisheng Feng[2], Kai Sun[3], Yuanping Yi[3], Zhibo Li[2*]

[1]Key Laboratory of Optic-electric Sensing and Analytical Chemistry for Life Science, MOE; Key Laboratory of Biochemical Analysis, Shandong Province, College of Chemistry and Molecular Engineering, Qingdao University of Science and Technology, Qingdao 266042, P. R. China, [2]Key Laboratory of Biobased Polymer Materials, Shandong Provincial Education Department, School of Polymer Science and Engineering, Qingdao University of Science and Technology, Qingdao 266042, P. R. China, [3]Institute of Chemistry, Chinese Academy of Sciences, Beijing 100190, P. R. China

***Correspondence:**
Zongxia Guo: zxguo@qust.edu.cn; Zhibo Li: zbli@qust.edu.cn

1 Experimental

1.1 Materials, Synthesis and characterization

All the reagents we used were of analytical reagent grade, and used without further purification.

Terrylene imides derivative (DUO-TDI) was synthesized based on the reported methods developed by prof. Klaus Müllen.(Guo et al., 2017; Nolde et al., 2006)

The synthetic routes of target compound were shown in **Scheme S1**.

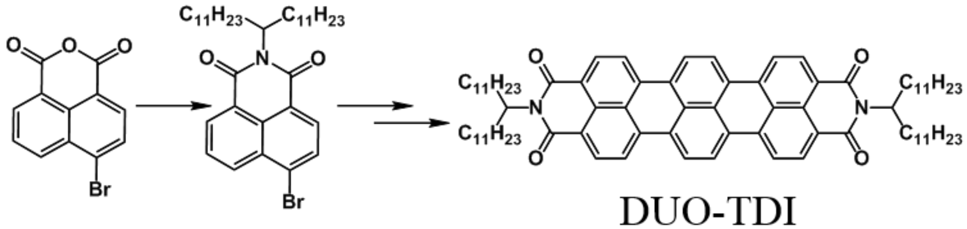

DUO-TDI

Scheme S1| Synthesis of DUO-TDI.

^{1}H NMR (400 MHz, THF-d_8, 25 °C): δ 8.14 (s, 4H), 8.01(m, 8H), 5.19 (m, 2H), 2.37 (m, 4H,), 1.93 (m, 4H), 1.45-1.27 (m, 40H), 0.87-0.83 (m, 12 H).

^{13}C NMR (100 MHz, THF-d_8, 25 °C): δ 164.12, 163.33, 134.98, 130.34, 129.55, 127.63, 125.51, 124.94, 123.30, 122.66, 121.79, 53.89, 32.99, 32.56, 31.60, 30.37, 29.99, 28.75, 27.86, 23.23, 14.66.

MALDI-TOF MS [M+] calculated for $C_{80}H_{106}N_2O_4$: 1158.82, found: 1159.03.

1.2 Sample preparation

At first, The DUO-TDI molecule powder was dissolved in tetrahydrofuran (THF) at room temperature to form stock solution, then, the stock solution was diluted to get self-assembly solutions of different concentration. Then, the stock solution was mixed with deionized water (H_2O) to the final concentration of 5.4×10^{-6} M. The solvent volume percent content of (deionized water, Vw, v%) was predesigned. In the following investigation, the deionized water content was continuously changed from 0 v% to 75 v%. Finally, all the resulting solutions were left at room temperature to fully self-assembly before further measurements.

1.3 Film preparation

Films on quartz plate and silicon wafer were prepared from a homogeneous THF solution (8.6×10^{-4} M). Films were dried before further measurements in a vacuum oven for 24 hours.

1.4 Measurements

NMR spectra were recorded on a Bruker AVANCE NEO 400 MHz NMR spectrometer (400 MHz for ^{1}H NMR and 100 MHz for ^{13}C NMR). Chemical shifts were reported in δ (ppm) and the residual deuterated solvent peak was used as reference. Matrix-assisted laser desorption/ionization time of flight mass spectrometry (MALDI-TOF MS) measurements were carried out on Bruker Microflex-LRF. UV-Vis absorption spectra were recorded using a HITACHI U-2910 UV-Vis absorption spectrometer. And the fluorescence spectra of solutions were carried out on a HITACHI F-2700 spectrometer. Atomic force microscopy (AFM) images were acquired from Bruker Multimode 8 Microscope with ScanAsyst Mode. AFM samples were prepared by dropping sample solutions on freshly stripped mica sheets and then dried in vacuum oven for at least 24 hours before measurements. FTIR spectra were measured using a Bruker VECTOR 33 FTIR spectrometer (Thermo Electron Corporation) by using the Attenuated Total Reflection (ATR) for powder sample, and solution samples were casted on KBr plate by using the Transmission method (TR). All STM experiments were performed at room temperature (20-25℃) using the MultiMode 8 system with the NanoScope V controller (Bruker). The STM images were obtained in the constant current mode. The STM tips used in this research were mechanically cut from a Pt/Ir wire (80/20, diameter 0.25 mm). The molecule DUO-TDI was dissolved in 1-octanoic acid (98%, Tokyo Chemical Industry) and 1-phenyloctane (98%, Tokyo Chemical Industry), respectively. The solutions were dropped onto freshly cleaved surface of Highly-Oriented Pyrolytic Graphites (HOPG, grade ZYB, Advanced Ceramics Inc, Cleveland, OH) for STM imaging. Detailed imaging conditions were given in the corresponding figure captions. The underneath graphite lattice was obtained by lowering the sample bias immediately after recording the monolayer images. Thermal gravimetric analyses (TGA) was

carried out on a TA Q20 by heating the samples from 30 to 800°C under nitrogen atmosphere at a heating rate of 10°C/min, samples were degassed at 120°C for 1h under vacuum before analysis. Differential scanning calorimetry (DSC) was performed on a TA instrument DSC 25.

1.5 Computational simulation

The optimization was performed using the Forcite module of Materials Studio 7.0. The DREIDING force field was implemented for the geometry optimizations (Mayo, 1990). HOMO and LUMO of the molecule were obtained using DMol3 module of Materials Studio 7.0. The initial supramolecular structures were inspired from the high-resolution STM images observed experimentally.

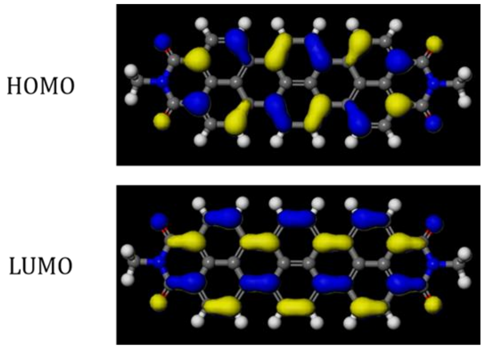

Figure S1 | Pictorial representations of HOMO and LUMO for DUO-TDI.

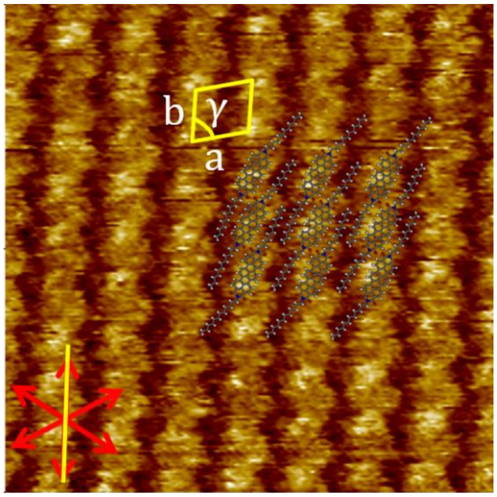

Figure S2 | High-resolution STM images (15×15 nm^2, 4.3×10^{-6} M) of DUO-TDI at the 1-Phenyloctane-HOPG interface. Imaging conditions I_{set} = 100 pA, V_{bias} = − 800 mV. The graphite symmetry axes are shown in red. The yellow lines indicate the directions of the unit-cell vectors. Molecular packing is indicated by models overlapping on the STM image.

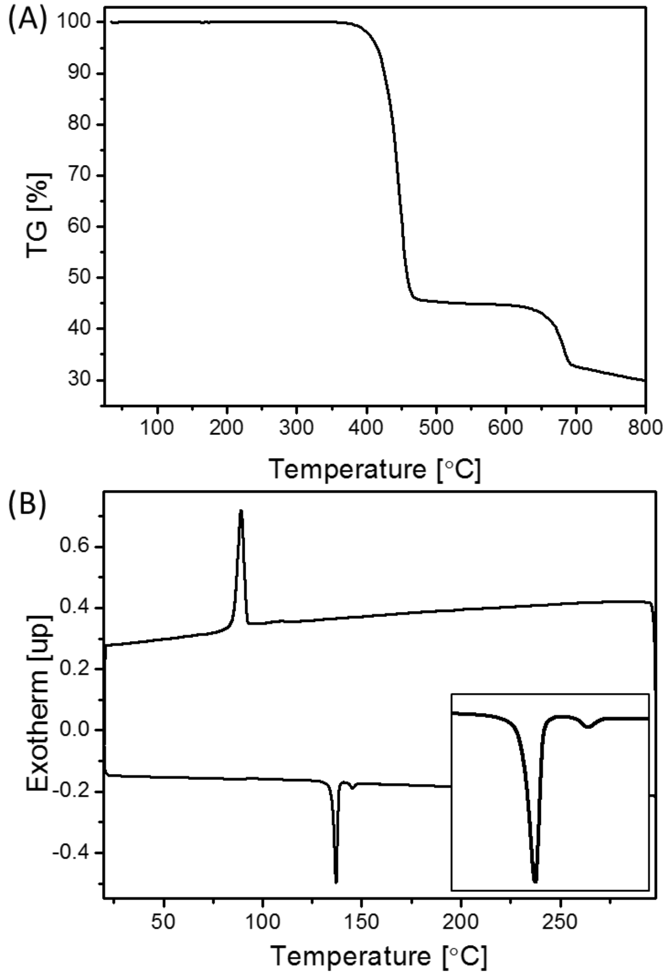

FIGURE S3 | (A) TGA and (B) DSC plots of DUO-TDI.

Reference

Guo, Z., Yu, P., Sun, K., Wang, W., Wei, Y., and Li, Z. (2017). Two-Dimensional Crystallization of Rylene Diimide Based n-Type Semiconductors Tuned by the Dimensions of the Aromatic Core at the Liquid-Solid Interface. *Chem. - Asian J.* 12(10)**,** 1104-1110. doi: 10.1002/asia.201700271.

Mayo, S. L. O., B. D.; Goddard, W. A. (1990). DREIDING: A Generic Force Field for Molecular Simulations. *J. Phys. Chem.* 94**,** 8897-8909. doi: 10.1021/j100389a010

Nolde, F., Pisula, W., Müller, S., Kohl, C., and Müllen, K. (2006). Synthesis and Self-Organization of Core-Extended Perylene Tetracarboxdiimides with Branched Alkyl Substituents. *Chem. Mat.* 18(16)**,** 3715-3725. doi: 10.1021/cm060742c.
